# Canadian Valuation of EQ-5D Health States: Preliminary Value Set and Considerations for Future Valuation Studies

**DOI:** 10.1371/journal.pone.0031115

**Published:** 2012-02-06

**Authors:** Nick Bansback, Aki Tsuchiya, John Brazier, Aslam Anis

**Affiliations:** 1 School of Population and Public Health, University of British Columbia, Vancouver, British Columbia, Canada; 2 Centre for Health Evaluation and Outcome Sciences, St Paul's Hospital, Vancouver, British Columbia, Canada; 3 Health Economics and Decision Sciences, School of Health and Related Research, University of Sheffield, Sheffield, United Kingdom; 4 Department of Economics, University of Sheffield, Sheffield, United Kingdom; Erasmus University Rotterdam, Netherlands

## Abstract

**Background:**

The EQ-5D is a preference based instrument which provides a description of a respondent's health status, and an empirically derived value for that health state often from a representative sample of the general population. It is commonly used to derive Quality Adjusted Life Year calculations (QALY) in economic evaluations. However, values for health states have been found to differ between countries. The objective of this study was to develop a set of values for the EQ-5D health states for use in Canada.

**Methods:**

Values for 48 different EQ-5D health states were elicited using the Time Trade Off (TTO) via a web survey in English. A random effect model was fitted to the data to estimate values for all 243 health states of the EQ-5D. Various model specifications were explored. Comparisons with EQ-5D values from the UK and US were made. Sensitivity analysis explored different transformations of values worse than dead, and exclusion criteria of subjects.

**Results:**

The final model was estimated from the values of 1145 subjects with socio-demographics broadly representative of Canadian general population with the exception of Quebec. This yielded a good fit with observed TTO values, with an overall R2 of 0.403 and a mean absolute error of 0.044.

**Conclusion:**

A preference-weight algorithm for Canadian studies that include the EQ-5D is developed. The primary limitations regarded the representativeness of the final sample, given the language used (English only), the method of recruitment, and the difficulty in the task. Insights into potential issues for conducting valuation studies in countries as large and diverse as Canada are gained.

## Introduction

Many difficult decisions in healthcare require value judgments. It is important to understand how society values different attributes of health to inform some of these decisions. Preference based instruments provide a classification of a respondent's health status and an empirically derived value, or preference, for that health state often from representative samples of the general population [1]. The preference for that health state can then be combined with duration to calculate outcomes such as Quality Adjusted Life Years (QALYs) [2]. While several preference based instruments are available, the EuroQol group's EQ-5D [3], [4], which describes health status by a combination of 5 attributes each comprised of 3 levels, is currently the most commonly used [5].

The first set of values for the EQ-5D health states was obtained from a sample of the general population in the United Kingdom in the early 1990s [6]. Since then, findings that peoples' health related preferences vary between countries [7] have led to several other population-based values [8], enabling policy makers to make informed decisions based on values from the population they serve. However, to date, no such values have been generated for Canada and consequently many studies have used population values from either the UK or US [9]–[17].

Conducting face to face interviews – the conventional method for eliciting public preferences - in a representative sample of the general adult population in Canada presents a number of logistical and resource limitation challenges. This study uses a conventional time trade-off (TTO) [18] exercise via a web survey in a sample of Canadians recruited from a market research panel and predicts values for all 243 EQ-5D health states conditional on the observed valuation data. The objective of this study is two-fold: to derive the first set of Canadian values of EQ-5D health states, and to provide insights into research designs for future valuation studies in large diverse countries such as Canada.

## Materials and Methods

### Ethics Statement

Ethical approval was obtained from the University of British Columbia behavioral ethics board. After being given detailed information, participants had to give written consent to begin the study.

### Survey design

The survey design was a quasi-replication of previous EQ-5D studies, using a protocol modified from the initial UK study [19]. The main differences from the original methodology include: a different selection of health states, a fewer number of health states valued by each participant, the use of a web survey instead of a face to face interview, no rank or visual analogue scale (VAS) exercise and lastly, recruitment via a market research panel. Reasons for these differences include multiple study objectives (the survey also included discrete choice experiment (DCE) questions based on the EQ-5D to study a methodological objective separate to the objective addressed in this paper) and resource limitations.

In total, 48 of the 243 possible EQ-5D health states were valued. This was based on a 36 item orthogonal array [20], supplemented with 12 further health states so that the 17 health states studied in nearly all previous EQ-5D surveys were included [21], [22]. With the constraints of the other tasks in the survey, pilot work suggested each respondent would be able to complete 5 different valuations in the time allocated. Consequently, the 48 health states were blocked into 12 sets using a computer algorithm so that each block was itself near orthogonal [20].

### Valuation procedure

**Figure 1:**
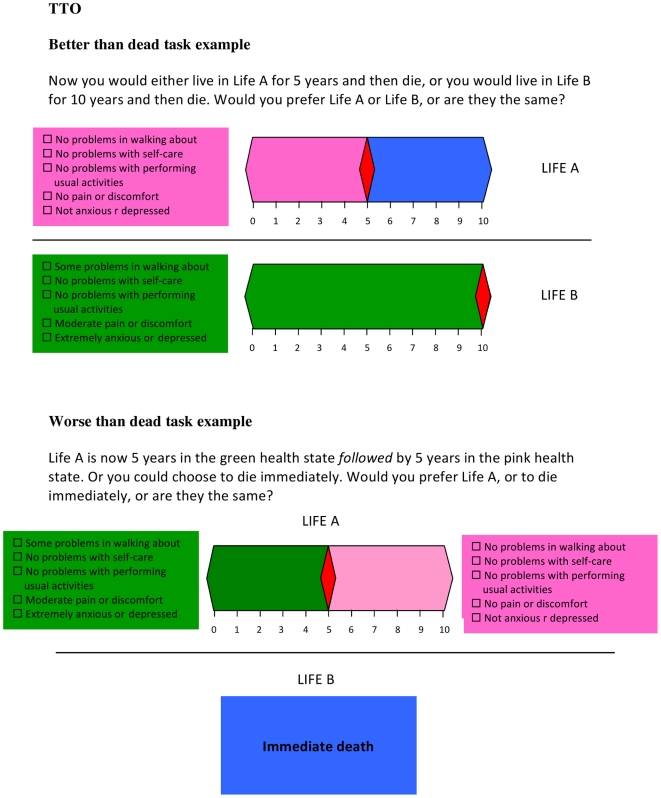
Example of the TTO task for better and worse than dead health states.

The TTO procedure required participants to first indicate whether the health state being valued was better or worse than dead (WTD). If the health state was considered better than dead, an iterative process was used where the respondent chose between living in the health state for 10-years or full health for *x* years. Changing *x*, the number of years in full health (beginning at 5 years and either increasing up to 10 years or decreasing to 0 years) to a point where the respondent was indifferent between the two choices, gave the value for the health state (*x*/10). A different procedure was used for states considered WTD whereby the choice was between immediate death, and spending a length of time (10−*x*) in the health state being valued followed by x years in full health. The value assigned to such health states was −*x*/(10−*x*). A visual prop (time board) was used to guide respondents (figure 1) [19]. Responses were measured in 3-month increments allowing the raw TTO scores *v* to range from 1 to -39. For consistency with most previous studies, values considered WTD (less than zero) were replaced by a monotonic transformation (where *v*′ = *v*/(1−*v*)) bounding values to −0.975 [23]. The alternative transformation for values WTD considered by Shaw (referred to as a linear transformation) was considered in a sensitivity analysis [24].

### Sampling framework

Members of a market research panel were invited to participate in the survey via email. Quota sampling was used to obtain a sample roughly representative of the age and gender of the Canadian general population. No incentive was provided specifically for participating in this survey, but participants in the panel that regularly completed surveys were offered various monthly and annual rewards.

Using previous EQ-5D studies as a guide [8], we considered including 1–2,000 respondents would obtain 5–10,000 valuations generating 25–50 valuations for each health state, sufficient to assess possible heterogeneity in preferences.

### Survey Structure

Individuals that accepted the email invitation to participate in the study were referred to a password-protected secure website that contained the survey. This presented information about the study, outlined the issues for consideration by completing the survey, and then gained consent. Respondents first described their own health using the EQ-5D descriptive system. After an introductory video, respondents were asked a series of questions including the 5 TTO tasks. The first TTO task included a logical test. Finally, respondents were asked to rate their difficulty in understanding and answering the TTO. Personal characteristics were not asked in the survey, but were obtained by the market research company for all invited individuals.

### Derivation of analytic sample

Respondents that failed to understand or engage with the TTO elicitation process were excluded from the primary analysis as their responses are not considered to represent their preferences. We used a variety of candidate criteria including: (i) the failure of a logical test, (ii) all 5 health states valued identically, (iii) multiple health states valued equal to 0.5, (iv) multiple health states valued as WTD, and (v) multiple logical inconsistencies between health state values (further information in Table 1). Since each of these criteria are subjective, we employed a previously described technique to determine the precise rules for inclusion [25]. This begins with a sample determined to have no problems (e.g. did not fail any of the *five* criteria under the most restrictive rules) and then added respondents based on iterative changes in each criteria (e.g. include respondents with 1 logical inconsistency) and tested whether there is evidence of systematic differences in the values obtained between the groups (new and old sample). This was repeated for all possible combination of criteria until the largest sample with no systematic differences in values is determined. Tests for systematic differences included: mean absolute difference (MAD) between each health states mean value; whether each of the 48 health states mean difference is statistically significantly different (using paired *t*-tests); the maximum difference between each mean profile value; the additional number of pairwise logical inconsistencies between mean profile values; the mean difference in the number of values WTD for each profile; and the maximum difference in the number of values WTD for each profile. A sensitivity analysis was undertaken using the whole sample.

**Table 1:** Problems with understanding and engaging with task from the 2033 respondents completing all five TTO tasks.

Criteria	Number of individuals (%)
i. Failed logical test	140 (7)
ii. Values for five health states identical	103 (5)*
iii. Number of tasks valued at 0.5	
0	1721 (85)
1	164 (8)
2	62 (3)
3	28 (1)
4	22 (1)
5 (all)	36 (2)
iv. Number of profiles valued worse than dead	
0	336 (17)
1	344 (17)
2	459 (23)
3	367 (18)
4	279 (14)*
5 (all)	248 (12)*
v. Number of pairwise logical inconsistencies	
0	850 (42)
1	519 (26)
2	303 (15)*
3	193 (9)*
4	97 (5)*
5	45 (2)*
6	24 (1)*
7+	2 (0)*
Total data problems*	888 (44)

*indicates final criteria used to determine respondents failing to engage or understand with the task. Further information available from the author.

Notes:

i. The logical test appears only in the first TTO task where respondents are asked if they would prefer 10 years in full health or 10 years in a health state worse than full health.

ii. Given the experimental design, the five scenarios given to each respondent included a mixture of mild and severe health states. If the respondent considers all five health states to have the same value then they were deemed to have not understood the task.

iii. For health states better than dead, the iteration procedure begins at 5 years (value of 0.5). The further away from 5 years the point of indifference is found, the more choices are required (and therefore more time). If the respondent was not engaged, the quickest way to complete the task is to answer at 5 years. Of course their true preference might be at 5 years, and so the number of consecutive values at 0.5 from the last TTO task are counted, as this might indicate whether they lost interest during the exercise (e.g. if values were 0.7, 0.3, 0.5, 0.5, 0.5 then this would be counted as 3, but 0.5, 0.5, 0.7, 0.8, 0.3 would be counted as zero).

iv. The first question in the task determines if the respondent considers the health state better or worse than dead. A number of health states considered worse than dead was considered to indicate an unengaged respondent.

v. A pairwise logical inconsistency was considered where the state with a less severe problem on a particular dimension, compared to another state, given its problems on the other dimensions are no more severe – e.g., 11121 versus 11131 and 32211 versus 32313 – is given a lower value.

### Statistical analysis

Descriptive statistics of the sample's characteristics were calculated. Comparisons between subgroups were made using t tests for interval data and χ^2^ tests for nominal data. Visual comparisons were made with characteristics of the Canadian general population using the Canadian Community Health Survey [26] and a previous EQ-5D study from the Canadian population [27].

TTO values were subtracted from 1 so that the dependent variable represents a measure of disutility, with a value of 1 equal to ‘immediate death’ and a value of 0 equal to full health. A random effect model was fitted using an additive specification [23], [28]. Various strategies were tested to account for interactions in the main effects. The N3 model assigns a dummy variable equal to 1 if any of the attributes was at level 3, and 0 otherwise [23]. The D1 model comprises of 4 terms: D1 represents the number of attributes with problems beyond the first and replaces the constant term; I2 represents the number of attributes at level 2 beyond the first; I2-squared is the square of I2; I3 represents the number of attributes at level 3 beyond the first, and I3-squared is the square of I3 [24].

The goodness-of-fit of models was assessed using: the square of the Pearson product-moment correlation between the observed and predicted health state values for each individual (R2), the mean absolute error (MAE) for predicting the mean observed values by health state, and the number of health states with prediction errors greater than 0.05 or 0.10 in absolute magnitude.

### Other analysis

Models were re-estimated using the linear transformation for values considered WTD, and using the whole sample instead of those defined to not have problematic TTO responses. Comparisons were also made with EQ-5D values obtained from the UK and US valuation surveys [23], [24].

All comparisons were explored using graphical means, the Pearson correlation, systematic differences identified by assessing the mean absolute difference (MAD), and number of states with a difference that was greater than 0.05 and 0.1.

## Results

### Sample characteristics

Of the 7482 subjects invited to participate in the survey, 2394 responded and consented (32.0%) to participate. A total of 2326 respondents began the TTO tasks (97.2% of those that began the survey), of which 2033 completed all 5 TTO tasks (87.4% of those that began the tasks). Of the 293 that failed to complete all the TTO tasks, 197 subjects did not complete even the first task.

A total of 888 (43.7%) respondents that completed all five TTO tasks were identified to have potentially failed to understand or engage with the task (see Table 1 for breakdown). The final inclusion criteria used were: not all values the same, three or fewer values considered WTD, and one or no pairwise logical inconsistencies (further details available from author). In total, 1145 respondents, or 56.3% of the 2033 that completed the TTO were included in the primary analysis.

The socio-demographic characteristics of respondents and non-respondents are shown in Table 2, along with Canadian general population statistics. It can be seen that the invited sample (groups I–IV) generally matched the Canadian general population (group V) with the exception of education (subjects with less than secondary education), and geography (substantially fewer subjects in predominantly French speaking Quebec). Respondents (groups I–III) tended to be older than non-respondents (group IV, p<0.001), which plausibly explains differences in education, household income and marital status. Subjects failing to complete all five TTO tasks (group III) were typically older (p<0.001) than those that did complete the tasks (groups I and II). Interestingly, there were fewer differences in profiles between respondents completing all tasks but identified to have failed to understand or engage with the task (group II) to those that did (group I). Exceptions were gender (more females had no problems (p = 0.009)), geography (p = 0.002), and problems in usual activities (p = 0.006).

**Table 2:** Study sample characteristics and comparison with Canadian general population.

							p-value	
	I. No Problems (n = 1145)	II. Problems (n = 888)	III. Non completers (n = 293)	IV. Non respondents (n = 5088)	V. General population†	I. vs II.	I+IIvsIII	I+II+III vs IV
Age, mean (range), n (%)	50.32 (18–99)	51.61 (18–92)	56.12 (18–85)	43.44 (18–94)	-	0.076	0.000	0.000
18–29	145 (13)	121 (14)	19 (6)	1168 (23)	(21)	0.061	0.000	0.000
30–39	165 (14)	96 (11)	26 (9)	1118 (22)	(18)			
40–49	227 (20)	162 (18)	45 (15)	1118 (22)	(22)			
50–59	226 (20)	189 (21)	60 (20)	763 (15)	(17)			
60–69	259 (23)	193 (22)	82 (28)	656 (13)	(11)			
70–79	109 (10)	111 (13)	56 (19)	254 (5)	(7)			
80+	14 (1)	16 (2)	5 (2)	11 (0)	(4)			
Gender, n (%)								
Male	531 (46)	464 (52)	131 (45)	2471 (49)	(49)	0.009	0.175	0.901
Female	614 (54)	424 (48)	162 (55)	2617 (51)	(51)			
Education, n (%)								
Less than secondary	3 (0)	5 (1)	2 (1)	66 (1)	(17)	0.496	0.681	0.000
Secondary graduate	243 (21)	196 (22)	67 (23)	1443 (28)	(17)			
Post-secondary	899 (79)	687 (77)	224 (76)	3579 (70)	(67)			
Household income, n (%)								
$15,000 or less	28 (2)	28 (3)	6 (2)	435 (9)	(6)	0.244	0.870	0.000
$15,000–$30,000	120 (10)	103 (12)	32 (11)	822 (16)	(13)			
$30,000–$50,000	246 (21)	215 (24)	67 (23)	1250 (25)	(20)			
$50,000–$80,000	360 (31)	277 (31)	98 (33)	1408 (28)	(27)			
$80,000 or more	360 (31)	246 (28)	81 (28)	919 (18)	(34)			
Not stated	31 (3)	19 (2)	9 (3)	254 (5)	-			
Marital status, n (%)								
Married	638 (56)	447 (50)	168 (57)	2129 (42)	(54)	0.085	0.082	0.000
Common-law	92 (8)	79 (9)	24 (8)	503 (10)	(11)			
Widow/separated/divorced	183 (16)	171 (19)	59 (20)	860 (17)	(12)			
Single	228 (20)	188 (21)	42 (14)	1570 (31)	(23)			
Not stated	4 (0)	3 (0)	0 (0)	26 (1)	-			
Province, n (%)								
Alberta	141 (12)	103 (12)	32 (11)	556 (11)	(11)	0.002	0.268	0.008
British Columbia	203 (18)	166 (19)	46 (16)	862 (17)	(13)			
Manitoba	63 (6)	46 (5)	13 (4)	199 (4)	(4)			
New Brunswick	18 (2)	26 (3)	5 (2)	99 (2)	(2)			
Newfld. and Labrador	9 (1)	20 (2)	7 (2)	109 (2)	(2)			
Nova Scotia	41 (4)	35 (4)	19 (6)	300 (6)	(3)			
Ontario	565 (49)	408 (46)	146 (50)	2498 (49)	(39)			
Prince Edward Island	2 (0)	13 (1)	0 (0)	30 (1)	(0)			
Quebec	59 (5)	40 (5)	12 (4)	233 (5)	(24)			
Saskatchewan	44 (4)	31 (3)	13 (4)	202 (4)	(3)			
EQ-5D attribute, n (%)								
Mobility								
Problems	256 (22)	184 (21)	62 (21)	-	(22)	0.374	0.851	-
No problems	889 (78)	704 (79)	231 (79)	-	(78)			
Self-care								
Problems	46 (4)	37 (4)	8 (3)	-	(4)	0.866	0.264	-
No problems	1099 (96)	851 (96)	285 (97)	-	(96)			
Usual activities								
Problems	259 (23)	157 (18)	54 (18)	-	(19)	0.006	0.418	-
No problems	886 (77)	731 (82)	239 (82)	-	(81)			
Pain/discomfort								
Problems	585 (51)	441 (50)	157 (54)	-	(44)	0.523	0.318	-
No problems	560 (49)	447 (50)	136 (46)	-	(56)			
Anxiety/depression								
Problems	356 (31)	247 (28)	74 (25)	-	(29)	0.109	0.121	-
No problems	789 (69)	641 (72)	219 (75)	-	(71)			
EQ-5D UK index, mean (SE)	0.8 (0.01)	0.82 (0.01)	0.82 (0.01)	-	-	0.166	0.228	-
EQ-5D US index, mean (SE)	0.85 (0)	0.86 (0.01)	0.86 (0.01)	-	-	0.143	0.309	-

† For non EQ-5D variable, from the Canadian Community Health Survey, 2006 [39]. Note, only % are relevant from such a survey, and specific ages not available. For EQ-5D variables, from Johnson and colleagues [40].

Of the individuals included in the final analysis, 88% (n = 1009) deemed the TTO task as not very or at all difficult to *understand*, while only 3 people found the task very difficult to *understand*. Some 50% (n = 571) found the task not very or at all difficult to *answer*, while 41% (n = 467) found it fairly difficult to *answer*. Interestingly, the difficulties in *answering* the task were not statistically different to the responses from the 888 individuals identified as potentially failing to understand or engage with the task, but difficulties in *understanding* the task were (with included individuals finding it easier as expected).

### Values

Amongst the main sample of 1145, on average there were over 97 values for each health state (range 74–185, with the exception of worst health state where values were obtained from all respondents). Mean values for each health state ranged from 0.885 (health state 21111) to −0.309 (33333) (Table 3).

**Table 3:** Observed and predicted values.

Health	Observed			Predicted	Difference
State*	N	Mean (SE)	Median (IQR)	Mean (SE)	
11112	86	0.853 (0.029)	0.990 (0.806, 1.000)	0.826 (0.020)	0.027
11113	122	0.575 (0.038)	0.625 (0.500, 0.888)	0.609 (0.020)	−0.034
11121	84	0.860 (0.019)	0.900 (0.800, 1.000)	0.844 (0.020)	0.016
11131	101	0.572 (0.036)	0.550 (0.500, 0.800)	0.591 (0.021)	−0.019
11133	96	0.263 (0.058)	0.375 (−0.119, 0.700)	0.311 (0.022)	−0.048
11211	87	0.828 (0.030)	0.950 (0.713, 1.000)	0.817 (0.020)	0.011
11222	99	0.766 (0.028)	0.825 (0.675, 0.997)	0.709 (0.022)	0.057
11312	93	0.696 (0.038)	0.700 (0.525, 0.990)	0.720 (0.023)	−0.024
12111	107	0.855 (0.019)	0.950 (0.788, 1.000)	0.819 (0.020)	0.036
12213	103	0.383 (0.055)	0.500 (0.338, 0.725)	0.466 (0.022)	−0.083
12231	91	0.557 (0.041)	0.500 (0.475, 0.850)	0.449 (0.023)	0.108
12321	96	0.622 (0.028)	0.625 (0.500, 0.806)	0.669 (0.024)	−0.047
12332	101	0.352 (0.047)	0.500 (0.256, 0.675)	0.352 (0.024)	0.000
13113	74	0.322 (0.063)	0.475 (0.206, 0.600)	0.385 (0.022)	−0.063
13122	99	0.508 (0.041)	0.513 (0.463, 0.750)	0.557 (0.024)	−0.049
13223	93	0.337 (0.055)	0.500 (0.075, 0.700)	0.268 (0.023)	0.069
13311	82	0.589 (0.038)	0.525 (0.500, 0.850)	0.560 (0.024)	0.029
21111	104	0.885 (0.017)	0.950 (0.869, 1.000)	0.843 (0.020)	0.042
21112	91	0.823 (0.022)	0.900 (0.725, 0.999)	0.780 (0.022)	0.043
21212	94	0.801 (0.020)	0.875 (0.700, 0.982)	0.708 (0.020)	0.093
21232	83	0.459 (0.050)	0.500 (0.200, 0.775)	0.411 (0.020)	0.048
21233	96	0.045 (0.061)	0.300 (−0.575, 0.500)	0.193 (0.021)	−0.148
21321	103	0.688 (0.033)	0.713 (0.500, 0.970)	0.693 (0.024)	−0.005
21333	91	0.113 (0.057)	0.288 (−0.375, 0.500)	0.160 (0.022)	−0.047
22112	104	0.714 (0.032)	0.775 (0.619, 0.950)	0.710 (0.023)	0.004
22123	87	0.526 (0.044)	0.625 (0.463, 0.788)	0.448 (0.024)	0.078
22131	93	0.489 (0.044)	0.525 (0.375, 0.775)	0.475 (0.023)	0.014
22222	96	0.585 (0.047)	0.613 (0.475, 0.900)	0.593 (0.020)	−0.008
22232	89	0.377 (0.047)	0.500 (0.200, 0.625)	0.340 (0.020)	0.037
22313	103	0.412 (0.048)	0.500 (0.300, 0.725)	0.387 (0.023)	0.025
23121	102	0.547 (0.041)	0.700 (0.406, 0.825)	0.575 (0.023)	−0.028
23222	115	0.448 (0.044)	0.525 (0.375, 0.725)	0.440 (0.021)	0.008
23231	86	0.155 (0.068)	0.200 (−0.425, 0.600)	0.250 (0.021)	−0.095
23232	88	0.179 (0.061)	0.275 (−0.188, 0.500)	0.187 (0.020)	−0.008
23233	104	−0.046 (0.060)	−0.075 (−0.675, 0.500)	−0.030 (0.020)	−0.016
23311	101	0.493 (0.045)	0.525 (0.400, 0.800)	0.514 (0.024)	−0.021
31211	102	0.576 (0.035)	0.625 (0.381, 0.825)	0.495 (0.022)	0.081
31221	100	0.435 (0.049)	0.500 (0.350, 0.775)	0.450 (0.023)	−0.015
31323	91	0.109 (0.066)	0.375 (−0.575, 0.500)	0.136 (0.022)	−0.027
32211	99	0.310 (0.053)	0.488 (0.175, 0.563)	0.424 (0.022)	−0.114
32223	93	0.160 (0.062)	0.400 (−0.500, 0.500)	0.099 (0.021)	0.061
32232	103	0.004 (0.061)	−0.050 (−0.625, 0.500)	0.063 (0.022)	−0.059
32313	86	0.073 (0.062)	0.113 (−0.544, 0.500)	0.111 (0.020)	−0.038
32323	86	0.025 (0.064)	0.025 (−0.500, 0.500)	0.066 (0.021)	−0.041
33213	92	−0.035 (0.058)	−0.225 (−0.506, 0.500)	−0.010 (0.021)	−0.025
33323	98	−0.121 (0.053)	−0.225 (−0.588, 0.500)	−0.087 (0.018)	−0.034
33332	85	−0.196 (0.041)	−0.425 (−0.675, 0.400)	−0.123 (0.019)	−0.073
33333	1145	−0.309 (0.016)	−0.500 (−0.725, 0.025)	−0.340 (0.013)	0.031
**MAE**					**0.044**

*each health state is described by the level for each attribute where ‘no health problems’ is denoted level 1, ‘moderate health problems’ level 2, and ‘severe health problems’ level 3.

The coefficients, model fit, and prediction statistics from the regression models based on 5725 observations are shown in Table 4. All the coefficients were statistically significant (p<0.01) and logically ordered with level 2 terms positive, and level 3 terms larger than level 2 for each attribute. When not including any interactions, the model fit resulted in an R^2^ of 0.403 and MAE of 0.044, similar to studies in the UK and US [19], [20]. Only three of the predicted 243 health state values differed to observed values by more than 0.1 (Table 3). While the addition of the N3 interaction term resulted in a statistically significant coefficient, it did not improve the model statistics. Only 2 of the D1 interaction terms were significant at the 5% level, and while there were minor improvements in MAE and R2, the number of health states with a difference in predicted versus observed value greater than 0.1 increased from three to five. We determined the final model to therefore not include any interactions, similar to previous studies in Japan [22], Denmark [29], and Zimbabwe [30]. Models were robust to the inclusion of socio-demographic variables, with the size of coefficients changing by less than 3 decimal points, and therefore no weighting was used to correct for non representativeness of the sample.

**Table 4:** Parameter estimates for random effects models including N3 and D1 terms (mean (SE)), and statistics relating to the comparison between observed and predicted values.

	Primary models† ‡			Sensitivity analysis	
	No interaction	Inclusion of N3	Inclusion of D1	All respondents†	Linear WTD with D1‡
Constant	0.111 (0.018)**	0.117 (0.018)**	-	0.487 (0.017)**	-
Mobility level 2	0.046 (0.015)**	0.045 (0.015)**	0.140 (0.022)**	0.020 (0.013)	0.144 (0.015)**
Mobility level 3	0.322 (0.018)**	0.319 (0.018)**	0.452 (0.034)**	0.176 (0.015)**	0.420 (0.019)**
Self-care level 2	0.071 (0.016)**	0.067 (0.016)**	0.159 (0.025)**	0.083 (0.013)**	0.162 (0.016)**
Self-care level 3	0.224 (0.016)**	0.206 (0.017)**	0.341 (0.027)**	0.177 (0.013)**	0.346 (0.016)**
Usual activities level 2	0.072 (0.017)**	0.061 (0.017)**	0.145 (0.025)**	0.106 (0.014)**	0.147 (0.017)**
Usual activities 3	0.105 (0.018)**	0.083 (0.020)**	0.201 (0.030)**	0.084 (0.016)**	0.251 (0.016)**
Pain/depression level 2	0.045 (0.016)**	0.050 (0.016)**	0.138 (0.025)**	0.043 (0.014)**	0.142 (0.016)**
Pain/depression level 3	0.298 (0.015)**	0.288 (0.016)**	0.421 (0.025)**	0.203 (0.013)**	0.392 (0.015)**
Anxiety/depression level 2	0.063 (0.016)**	0.060 (0.016)**	0.168 (0.023)**	0.030 (0.014)*	0.159 (0.014)**
Anxiety/depression level 3	0.280 (0.016)**	0.256 (0.018)**	0.393 (0.025)**	0.202 (0.014)**	0.370 (0.015)**
N3	-	0.061 (0.022)**	-		
D1	-	-	−0.072 (0.028)*		−0.110 (0.014)**
I2	-	-	−0.042 (0.040)		-
I2^2^	-	-	0.004 (0.006)		-
I3	-	-	−0.007 (0.035)		−0.043 (0.021)*
I3^2^	-	-	−0.012 (0.005)*		−0.012 (0.003)**
n	5725	5725	5725	10165	5725
R^2^	0.403	0.404	0.402	0.201	0.403
No. states differing by >|0.05|	14	16	11	44	7
No. states differing by >|0.1|	3	4	5	40	0
MAE	0.044	0.045	0.035	0.245	0.027

† Monotonic transformation of health states WTD.

‡ exclusion of respondents deemed to not have understood of engaged with the TTO task.

*significant at the 5% level.

**significant at the 1% level.

### Sensitivity analysis

**Figure 2:**
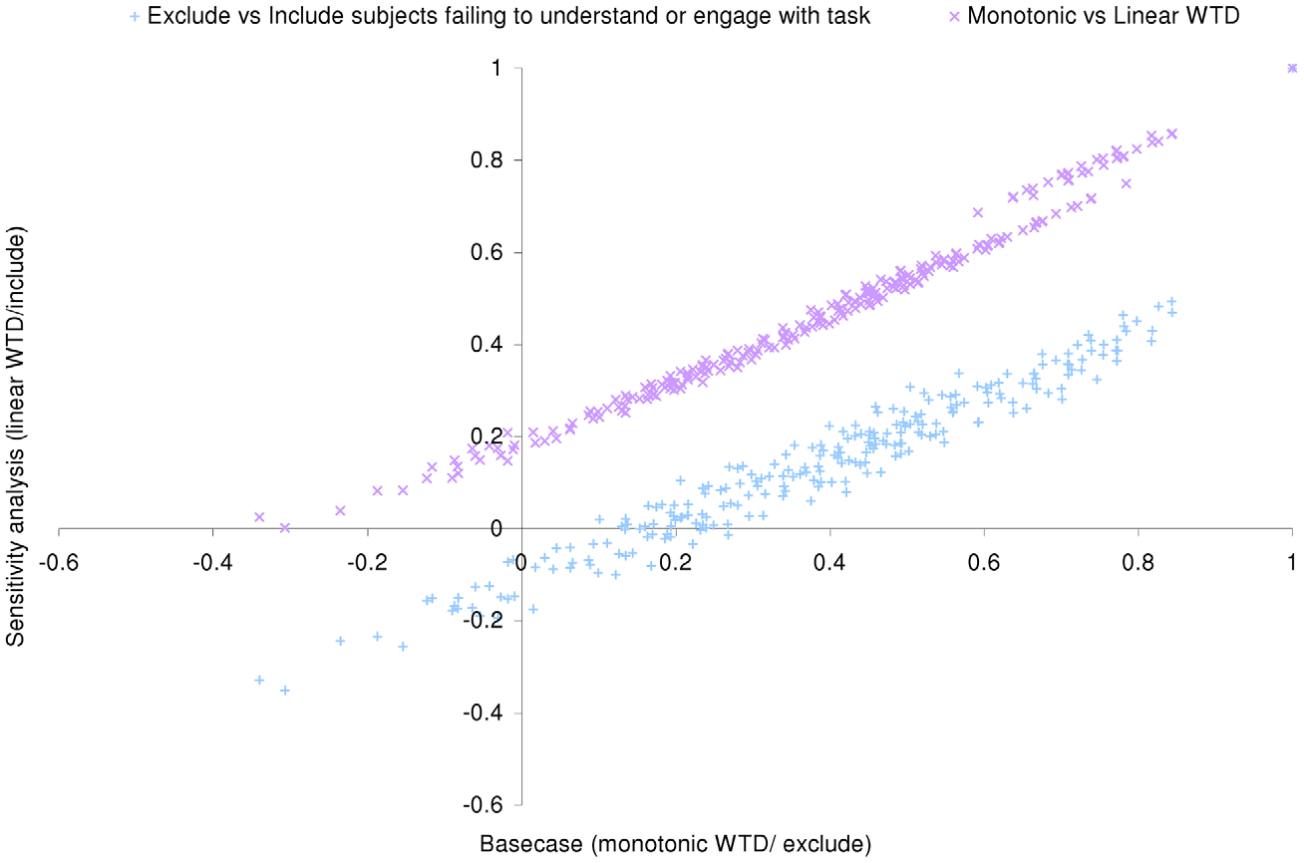
Sensitivity analysis – comparison between predicted values from the primary model with (i) those using the full sample and (ii) using a linear WTD transformation.

The inclusion of respondents deemed to not engage or understand the TTO task modified the coefficient estimates substantially. In particular, the constant increased from 0.111 to 0.487, which means the values for mild health states are dramatically different (e.g. for health state 21111 the value is 0.493 versus 0.843 in the main model). Figure 2 compares the 243 predicted health state values between the two sets of respondents demonstrating the systematic differences (MAD = 0.240, n>|0.05| = 236, n>|0.10| = 225).

Figure 2 also compares the values for only the main sample when health states WTD were transformed using the linear and monotonic methods. As with previous findings [24], we found the choice of transformation to impact results substantially (MAD = 0.085, n>|0.05| = 159, n>|0.10| = 79).

### Comparison to values from other countries

**Figure 3:**
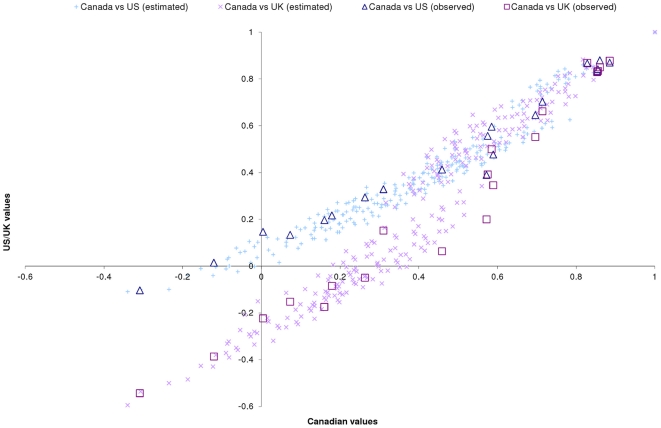
Comparison of observed and predicted values from Canada with those in the UK and US.

Figure 3 is a scatter plot comparing the 243 predicted health states between the present study and from the previous EQ-5D studies in the US and the UK [23], [24]. While there is a high correlation between the predicted values (Pearson's rho = 0.964 and 0.963 for the US and UK respectively), it appears that Canadian values are systematically different to both the US and UK values, placing lower values on severe health states in comparison to the US (MAD = 0.057, n>|0.05| = 121, n>|0.10| = 37) and placing higher values on severe health states in comparison to the UK (MAD = 0.169, n>|0.05| = 178, n>|0.10| = 137). The figure also shows this pattern is also found in comparisons between the 20 common observed health states and so these differences do not appear to be an artifact of different model specifications. As explored above, the differences in US values could be attributable to the linear transformation of health states WTD.

## Discussion

This is the first study to provide a population-based set of values for EQ-5D health states in Canada. Coefficients were logically ordered and model fit was similar to other studies, but final values differed from previous value sets in the US and UK meaning previous economic evaluations using the EQ-5D may not provide accurate information to Canadian decision makers. Researchers can apply these values to studies collecting the EQ-5D to generate QALYs based on Canadian preferences (Tables S1 and S2).

While an objective of the study was to broadly follow previous country valuations, conducting such surveys in a country such as Canada provides unique challenges for both recruitment and administration which can influence the representativeness of values and the comparability of results with other country studies. Another important objective of this study was to identify issues for researchers conducting valuation studies in Canada and the potential influence of these issues on values and resources.

Our decision to use a web panel maintained by a market research company in a survey conducted in the English language may have affected the representativeness of the invited sample in comparison to the Canadian general population. Not all (only 80%) Canadians have access to the internet [31]. Moreover, subjects in market research panels may have a greater familiarity with understanding survey questions. Finally, the language this study was conducted in – English – is the preferred language for only 67% of Canadians [32].

These limitations however should be compared with alternative designs. Face-to-face interviews could overcome some of the highlighted problems, but conducting interviews in a country as large as Canada, in particular in rural areas, would require resources many times greater than those required for this study. Recruitment to such a study would be limited by the number of people who do not have publicly listed landline telephones [33]. Such a design would also not be able to compare socio-demographics of non-responders as we were able in this study. Whether such additional resources are worth the improvement in representativeness are debatable. Values for the Health Utilities Index [34], the predominant valuation study in Canada interviewed subjects from only one city, whereas the CLAMES survey only included 146 participants [35]. In contrast, this study included over 1000 respondents from all ten provinces including rural areas. Including a French version would improve representativeness. However, care should be given to ensure an accurate translation of the survey (in addition to the already existing Canadian French version of EQ-5D) and caution to the design and analysis to ensure values from the two versions can be combined appropriately.

Using a computer to elicit values instead of face-to-face interviews also may impact the comparability of the values to previous studies [36]. A computer based TTO has been used in previous EQ-5D studies [29], but often at an interview where assistance is available and not via the web. The advantages of a computer based TTO include the potential to reduce interviewer bias, errors in question routing and data input, and easy randomization of question ordering. Disadvantages of using a computer via the web are that engagement and understanding of the TTO task appears to suffer. This study found that 12.6% did not finish all the TTO tasks, and then excluded 43.7% of the respondents from the final models due to concerns over engagement or understanding. Previous EQ-5D studies, while using different exclusion criteria, have excluded between 7% [24] and 57% [25] of respondents from final models. It is reasonable to conjecture that the use of the web partially explains these differences. There is a strong argument to exclude values from respondents that appeared to fail to understand or engage with the TTO task since their responses do not represent their preferences, however since it is important to use representative samples in final models, including the elderly and low educated, any exclusion is in itself problematic. Looking for alternative elicitation methods such as rankings [37] or DCEs [38], [39] which may be simpler for subjects to understand should be explored.

The final issue regards how values for health states respondents considered WTD are interpreted. Our results find that the choice of method influences the values, similar to other findings. Unfortunately, the choice of method is arbitrary [40]. Our primary results employ the most commonly used method, enabling a more fair comparison with previous studies. The main values from this study should be cautiously compared to those derived in the US which used a different method for transforming values considered WTD. The consequence is that depending on which method is chosen, QALY gains of different size would be generated and policy-makers might be faced with opposing conclusions based on the choices. This highlights the importance of developing elicitation methods not requiring subjective transformations. Work on the ‘lead time’ TTO [41], [42] and the DCE [38], [39] appear to be promising alternatives in early development.

In conclusion, this study provides estimates for developing QALYs based on the EQ-5D using preferences from a broadly representative sample of the Canadian population with the exception of Quebec. With the resources available for this study, we conclude that the use of the internet and a market research panel is the preferred method for generating values to be used by policymakers in countries as large and diverse as Canada in comparison to alternative design. Limitations with the design remain, and we suggest a focus on cognitively easier methods that enable more respondents to engage and understand the tasks. Including a French version of the survey, and overcoming issues with the interpretation of health states considered worse than dead would also further improve future designs. Focus should be given to these limitations before the valuation of the 5-level EQ-5D commences [43]. Until these limitations can be addressed, the value set provided in this study offers substantial improvements over using preferences from the UK or US in the Canadian context for the EQ-5D.

## Supporting Information

Table S1 **Example of scoring algorithm.**
(DOC)

Table S2 **List of values for all health states.**
(DOC)
