# Supplementary material for: Canadian Valuation of EQ-5D Health States: Preliminary Value Set and Considerations for Future Valuation Studies
Source: PLoS One. 2012 Feb 6;7(2):e31115. doi: 10.1371/journal.pone.0031115 (PMC3273479; doi:10.1371/journal.pone.0031115)
Supplement: Table S1 — Example of scoring algorithm. (DOC) [file pone.0031115.s001.doc]

**Supporting Information**

**Table S1: Example of scoring algorithm**

| Health State 12213 |
| --- |
| Full health = 1.000 |
| Constant term (for any dysfunctional health state) (subtract 0.111) |
| Mobility: level 1 (subtract 0) |
| Self-care: level 2 (subtract 0.071) |
| Usual activities: level 2 (subtract 0.072) |
| Pain/discomfort: level 1 (subtract 0) |
| Anxiety/depression: level 3 (subtract 0.280) |

Hence, the predicted value for state 11223 is

1.000 – 0.111 - 0.000 - 0.071 - 0.072 – 0.000 - 0.280 = 0.466
