# Supplementary material for: Canadian Valuation of EQ-5D Health States: Preliminary Value Set and Considerations for Future Valuation Studies
Source: PLoS One. 2012 Feb 6;7(2):e31115. doi: 10.1371/journal.pone.0031115 (PMC3273479; doi:10.1371/journal.pone.0031115)
Supplement: Table S2 — List of values for all health states. (DOC) [file pone.0031115.s002.doc]

**Supporting Information**

**Table S2: List of values for all health states**

| **Health state** | **Value** | **Health state** | **Value** | **Health state** | **Value** |
| --- | --- | --- | --- | --- | --- |
| 11111 | 1.000 | 21111 | 0.843 | 31111 | 0.567 |
| 11112 | 0.826 | 21112 | 0.780 | 31112 | 0.504 |
| 11113 | 0.609 | 21113 | 0.563 | 31113 | 0.286 |
| 11121 | 0.844 | 21121 | 0.799 | 31121 | 0.522 |
| 11122 | 0.781 | 21122 | 0.736 | 31122 | 0.459 |
| 11123 | 0.564 | 21123 | 0.518 | 31123 | 0.242 |
| 11131 | 0.591 | 21131 | 0.546 | 31131 | 0.269 |
| 11132 | 0.528 | 21132 | 0.483 | 31132 | 0.206 |
| 11133 | 0.311 | 21133 | 0.265 | 31133 | -0.011 |
| 11211 | 0.817 | 21211 | 0.771 | 31211 | 0.495 |
| 11212 | 0.754 | 21212 | 0.708 | 31212 | 0.432 |
| 11213 | 0.537 | 21213 | 0.491 | 31213 | 0.214 |
| 11221 | 0.772 | 21221 | 0.727 | 31221 | 0.450 |
| 11222 | 0.709 | 21222 | 0.664 | 31222 | 0.387 |
| 11223 | 0.492 | 21223 | 0.446 | 31223 | 0.170 |
| 11231 | 0.519 | 21231 | 0.474 | 31231 | 0.197 |
| 11232 | 0.456 | 21232 | 0.411 | 31232 | 0.134 |
| 11233 | 0.239 | 21233 | 0.193 | 31233 | -0.083 |
| 11311 | 0.784 | 21311 | 0.738 | 31311 | 0.461 |
| 11312 | 0.720 | 21312 | 0.675 | 31312 | 0.398 |
| 11313 | 0.503 | 21313 | 0.458 | 31313 | 0.181 |
| 11321 | 0.739 | 21321 | 0.693 | 31321 | 0.417 |
| 11322 | 0.676 | 21322 | 0.630 | 31322 | 0.354 |
| 11323 | 0.459 | 21323 | 0.413 | 31323 | 0.136 |
| 11331 | 0.486 | 21331 | 0.440 | 31331 | 0.164 |
| 11332 | 0.423 | 21332 | 0.377 | 31332 | 0.101 |
| 11333 | 0.206 | 21333 | 0.160 | 31333 | -0.117 |
| 12111 | 0.819 | 22111 | 0.773 | 32111 | 0.496 |
| 12112 | 0.755 | 22112 | 0.710 | 32112 | 0.433 |
| 12113 | 0.538 | 22113 | 0.493 | 32113 | 0.216 |
| 12121 | 0.774 | 22121 | 0.728 | 32121 | 0.452 |
| 12122 | 0.711 | 22122 | 0.665 | 32122 | 0.389 |
| 12123 | 0.494 | 22123 | 0.448 | 32123 | 0.171 |
| 12131 | 0.521 | 22131 | 0.475 | 32131 | 0.199 |
| 12132 | 0.458 | 22132 | 0.412 | 32132 | 0.136 |
| 12133 | 0.241 | 22133 | 0.195 | 32133 | -0.082 |
| 12211 | 0.746 | 22211 | 0.701 | 32211 | 0.424 |
| 12212 | 0.683 | 22212 | 0.638 | 32212 | 0.361 |
| 12213 | 0.466 | 22213 | 0.420 | 32213 | 0.144 |
| 12221 | 0.702 | 22221 | 0.656 | 32221 | 0.380 |
| 12222 | 0.639 | 22222 | 0.593 | 32222 | 0.316 |
| 12223 | 0.421 | 22223 | 0.376 | 32223 | 0.099 |
| 12231 | 0.449 | 22231 | 0.403 | 32231 | 0.127 |
| 12232 | 0.386 | 22232 | 0.340 | 32232 | 0.063 |
| 12233 | 0.168 | 22233 | 0.123 | 32233 | -0.154 |
| 12311 | 0.713 | 22311 | 0.668 | 32311 | 0.391 |
| 12312 | 0.650 | 22312 | 0.604 | 32312 | 0.328 |
| 12313 | 0.433 | 22313 | 0.387 | 32313 | 0.111 |
| 12321 | 0.669 | 22321 | 0.623 | 32321 | 0.346 |
| 12322 | 0.605 | 22322 | 0.560 | 32322 | 0.283 |
| 12323 | 0.388 | 22323 | 0.343 | 32323 | 0.066 |
| 12331 | 0.416 | 22331 | 0.370 | 32331 | 0.093 |
| 12332 | 0.352 | 22332 | 0.307 | 32332 | 0.030 |
| 12333 | 0.135 | 22333 | 0.090 | 32333 | -0.187 |
| 13111 | 0.665 | 23111 | 0.620 | 33111 | 0.343 |
| 13112 | 0.602 | 23112 | 0.557 | 33112 | 0.280 |
| 13113 | 0.385 | 23113 | 0.339 | 33113 | 0.063 |
| 13121 | 0.621 | 23121 | 0.575 | 33121 | 0.298 |
| 13122 | 0.557 | 23122 | 0.512 | 33122 | 0.235 |
| 13123 | 0.340 | 23123 | 0.295 | 33123 | 0.018 |
| 13131 | 0.368 | 23131 | 0.322 | 33131 | 0.045 |
| 13132 | 0.304 | 23132 | 0.259 | 33132 | -0.018 |
| 13133 | 0.087 | 23133 | 0.042 | 33133 | -0.235 |
| 13211 | 0.593 | 23211 | 0.548 | 33211 | 0.271 |
| 13212 | 0.530 | 23212 | 0.484 | 33212 | 0.208 |
| 13213 | 0.313 | 23213 | 0.267 | 33213 | -0.010 |
| 13221 | 0.549 | 23221 | 0.503 | 33221 | 0.226 |
| 13222 | 0.485 | 23222 | 0.440 | 33222 | 0.163 |
| 13223 | 0.268 | 23223 | 0.223 | 33223 | -0.054 |
| 13231 | 0.296 | 23231 | 0.250 | 33231 | -0.027 |
| 13232 | 0.232 | 23232 | 0.187 | 33232 | -0.090 |
| 13233 | 0.015 | 23233 | -0.030 | 33233 | -0.307 |
| 13311 | 0.560 | 23311 | 0.514 | 33311 | 0.238 |
| 13312 | 0.497 | 23312 | 0.451 | 33312 | 0.174 |
| 13313 | 0.279 | 23313 | 0.234 | 33313 | -0.043 |
| 13321 | 0.515 | 23321 | 0.470 | 33321 | 0.193 |
| 13322 | 0.452 | 23322 | 0.407 | 33322 | 0.130 |
| 13323 | 0.235 | 23323 | 0.189 | 33323 | -0.087 |
| 13331 | 0.262 | 23331 | 0.217 | 33331 | -0.060 |
| 13332 | 0.199 | 23332 | 0.154 | 33332 | -0.123 |
| 13333 | -0.018 | 23333 | -0.064 | 33333 | -0.340 |
